# Anti-Oxidative, Anti-Tumor-Promoting, and Anti-Carcinogensis Activities of Nitroastaxanthin and Nitrolutein, the Reaction Products of Astaxanthin and Lutein with Peroxynitrite

**DOI:** 10.3390/md10061391

**Published:** 2012-06-18

**Authors:** Takashi Maoka, Harukuni Tokuda, Nobutaka Suzuki, Hideaki Kato, Hideo Etoh

**Affiliations:** 1 Research Institute for Production Development, 15 Shimogamo-morimoto-cho, Sakyo-ku, Kyoto 606-0805, Japan; 2 Department of Complementary and Alternative Medicine, Clinical R&D, Graduate School of Medical Science, Kanazawa University, 13-1 Takara-machi, Kanazawa 920-8640, Japan; Email: htokuda@med.kanazawa-u.ac.jp (H.T.); pcam@med.kanazawa-u.ac.jp (N.S.); 3 Faculty of Agriculture, Shizuoka University, 836 Ohya, Suruga-ku, Shizuoka 422-8529, Japan; Email: hideakick7@hotmail.com (H.K.); acheto@ipc.shizuoka.ac.jp (H.E.)

**Keywords:** carotenoid, 15-nitroastaxanthin, 15-nitrolutein, anti-carcinogensis activity, anti-oxidative activity

## Abstract

Astaxanthin captured peroxynitrite to form nitroastaxanthins. 15-Nitroastaxanthin was a major reaction product of astaxanthin with peroxynitrite. Here, the anti-oxidative, anti-tumor-promoting, and anti-carcinogensis activities of 15-nitroastaxanthin were investigated. In addition to astaxanthin, 15-nitroastaxanthin showed excellent singlet oxygen quenching activity. Furthermore, 15-nitroastaxanthin showed inhibitory effects of *in vitro* Epstein-Barr virus early antigen activation and two-stage carcinogensis on mouse skin papillomas. These activities were slightly higher than those of astaxanthin. Similar results were obtained for the 15-nitrolutein, a major reaction product of lutein with peroxynitrite.

## 1. Introduction

Carotenoids have an excellent capacity to quench singlet oxygen (^1^O_2_) and inhibit lipid peroxidation. However, they cannot scavenge superoxide anion radicals (O_2_^−^), hydroxy radicals (OH·), or hydrogen peroxide (H_2_O_2_). On the other hand, there have been few reports on scavenging and/or the reaction of reactive nitrogen species with carotenoids [1]. Peroxynitrite (ONOO^−^), a reactive nitrogen species, is formed from superoxide (O_2_^−^) and nitric oxide (NO·) *in vivo*, and is a highly reactive oxidant that causes nitration of the aromatic ring of free tyrosine and protein tyrosine residues [2]. Furthermore, peroxynitrite was found to induce various forms of oxidative damage such as low-density lipoprotein (LDL) oxidation, lipid peroxidation, and DNA strand breakage [2].

Astaxanthin is a marine carotenoid, which is distributed in marine bacteria, algae, crustaceans, and fish. Astaxanthin is an excellent antioxidant and shows biological functions including anti-carcinogensis, anti-inflammatory, anti-diabetic, and anti-obesity activities [1]. Astaxanthin also inhibits reactive nitrogen species-induced inflammation [3]. Previously, we studied the reaction of astaxanthin with peroxynitrite *in vitro* and reported the formation of nitroastaxanthins by the reaction of astaxanthin with peroxynitrite [4,5]. This result suggested that astaxanthin could scavenge peroxynitrite to uptake peroxynitrite into its molecule by the formation of nitroastaxanthins. Lutein, a major carotenoid in green algae and higher plants, also showed radical scavenging, anti-carcinogenic, anti-diabetic, and anti-obesitic activities [1]. Lutein also formed nitroluteins and lutein-oxazine by the reaction with peroxynitrite *in vitro* [6]. Therefore, we assumed that astaxanthin and lutein could inhibit tyrosine nitration induced by peroxynitrite and that nitroastaxanthin and nitrolutein themselves may have ^1^O_2_ quenching and anti-carcinogensis activities.

**Figure 1:**
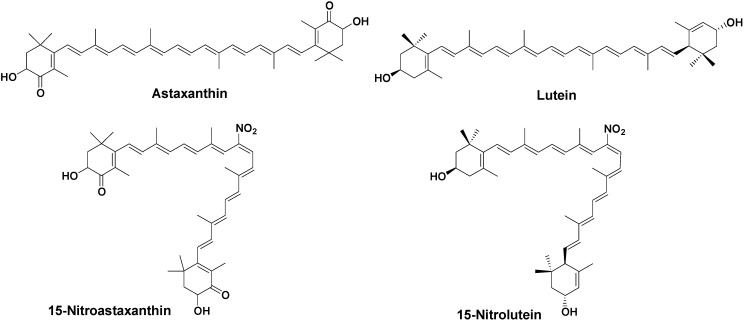
Structures of carotenoids and nitrocarotenoids.

In this study, we investigated the inhibitory effect of tyrosine nitration with peroxynitrite by astaxanthin and lutein. Furthermore, ^1^O_2_ quenching, anti-tumor-promoting, and anti-carcinogensis activities of 15-nitroastaxanthin and 15-nitrolutein (Figure 1), the major reaction products of astaxanthin and lutein with peroxynitrite, were studied.

## 2. Results and Discussion

### 2.1. Inhibition of Tyrosine Nitration with Peroxynitrite by Astaxanthin and Lutein

Peroxynitrite induces nitration of the aromatic ring of free tyrosine and protein tyrosine residues and causes various diseases [2].

Astaxanthin suppressed the formation of nitrotyrosine to about 10.0% of that of the control group. This effect was almost the same as that of γ-tocopherol (9.2%). A similar result was also obtained in the case of lutein (12.5%) These results indicate that astaxanthin and lutein are able to capture peroxynitrite to form nitroastaxanthin and nitrolutein and inhibit the tyrosine nitration.

It was reported that phenolic compounds such as *p*-coumaric acid, pelargonidin [7,8,9], and tocopherols [10] could scavenge peroxynitrite by the formation of nitro-*p*-coumaric acid, nitro-pelargonidin, and nitro-tocopherols, respectively, and protect against tyrosine nitration. Capsanthin and fucoxanthin also inhibited the nitration of tyrosine to form nitrocarotenoids [11]. These results indicated that carotenoids, with long polyene chains, could inhibit the tyrosine nitration to form nitrocarotenoids.

Mechanism of nitration of carotenoid with proxynitrite is uncertain. However, we assumed that this reaction might be a radical addition of nitroradical (NO_2_) with carotenoid.

It was reported that NO_2_ was formed from proxynitrite as following mechanism [12].

ONOO^−^ + ONOOH→ONOO^−^ + ·NO_2_ + OH^−^

Therefore, we assumed that ·NO_2_, generated from proxynitrite, took out hydrogen from carotenoid to form carotenoid radical. Then, NO_2_ reacted with carotenoid radical to form nitrocarotenoid. Carotenoids might have higher reactivity with proxynitrite and/or NO_2_ than phenol compounds such as tyrosine. Therefore, carotenoid could inhibit nitration of tyrosine.

### 2.2. Quenching Effects of ^1^O_2_ by 15-Nitroastaxanthin and 15-Nitrolutein

^1^O_2_ quenching activity of nitrocarotenoids was examined by the methylene blue-sensitized photooxidation method [13,14,15]. The IC_50_ values of ^1^O_2_ quenching activity were as follows; astaxanthin 7.0 μM, 15-nitroastaxanthin 20.0 μM (Table 1), and β-carotene 100 μM [15]. As well as astaxanthin, 15-nitroastaxanthin also showed excellent ^1^O_2_ quenching activity. This activity was slightly weaker than that of astaxanthin and higher than that of β-carotene. It is reported that carotenoids with more than eleven conjugated double bonds show excellent quenching activity for ^1^O_2_ and that the ^1^O_2_ quenching activity of carotenoids depends on the number of conjugated double bonds, polyene chain structures and functional groups [13,14,15,16,17]. 15-Nitroastaxanthin has the same conjugated double bonds system as that of astaxanthin. Therefore, 15-nitroastaxanthin exhibited strong ^1^O_2 _quenching activity. 15-Nitrolutein also showed ^1^O_2_quenching activity (IC_50_ value 71.5 μM). This activity was slightly weaker than that of lutein (IC_50_ value 60.5 μM) and higher than that of β-carotene (100 μM). These results indicated that not only carotenoids, but also nitro-carotenoids, could scavenge ^1^O_2_.

Concerning the ^1^O_2_ quenching activity of carotenoids [13,14,15,16,17], astaxanthin and 15-nitroastaxanthin showed strong activity and lutein and 15-nitrolutein exhibited moderate activity among the carotenoids ^1^O_2_ quenching activity investigated.

**Table 1:** ^1^O_2_quenching activity (%) ^a^ of carotenoids and nitrocarotenoids.

Concentration\Compounds	100 (μM)	10 (μM)	1 (μM)	IC_50_ (μM)
Astaxanthin	93.8	65.5	30.8	7.0
15-Nitroastaxanthin	91.8	48.5	5.0	20.0
Lutein	75.8	38.5	1.0	60.5
15-Nitrolutein	72.2	36.5	0.8	71.5

^a^ Triplicate assays were performed for each compound in this assay system.

### 2.3. Anti-Tumor-Promoting and Anti-Carcinogensis Activities of 15-Nitroastaxanthin and 15-Nitrolutein

The *in vitro* anti-tumor-promoting activity of 15-nitroastaxanthin and 15-nitrolutein was examined using an Epstein-Barr virus early antigen (EBV-EA) activation assay in Raji cells [18,19]. This assay was used to estimate the anti-tumor-promoting effects of several natural carotenoids [18,19]. The results are shown in Table 2. Both 15-nitroastaxanthin and 15-nitrolutein showed inhibitory effects on the EBV-EA induction of Raji cells without significant cytotoxicity (more than 60% viability of Raji cells) in this assay. 15-Nitroastaxanthin showed slightly higher activity than astaxanthin. Furthermore, 15-nitrolutein also showed slightly higher activity than lutein. These results showed that nitrocarotenoids themselves have anti-tumor-promoting activity.

**Table 2:** Relative rate of Epstein-Barr virus early antigen (EBV-EA) activation ^a^ with respect to the positive control (100%) in the presence of carotenoids and nitrocarotenoids.

Concentration (mol ratio/TPA) ^b^	1000	500	100	10	IC_50_ (nmol)
Compounds			values		
Astaxanthin	5.0 (70) ^c^	29.0	78.0	100	307
15-Nitroastaxanthin	4.1 (60) ^c^	28.5	76.9	100	300
Lutein	2.9 (70) ^c^	24.5	75.4	97.6	283
15-Nitrolutein	1.6 (60) ^c^	23.3	74.1	95.4	277

^a^ Values represent the percentage relative to the positive control values (100%). Triplicate assays were performed for each compound in this assay system; ^b^ TPA concentration was 20 ng (32 pmol); ^c^ Values in parentheses are percentage viability of Raji cells; There were significant differences (*P* < 0.01) in the carotenoids and nitrocarotenoids treatment groups compared to the control group.

**Figure 2:**
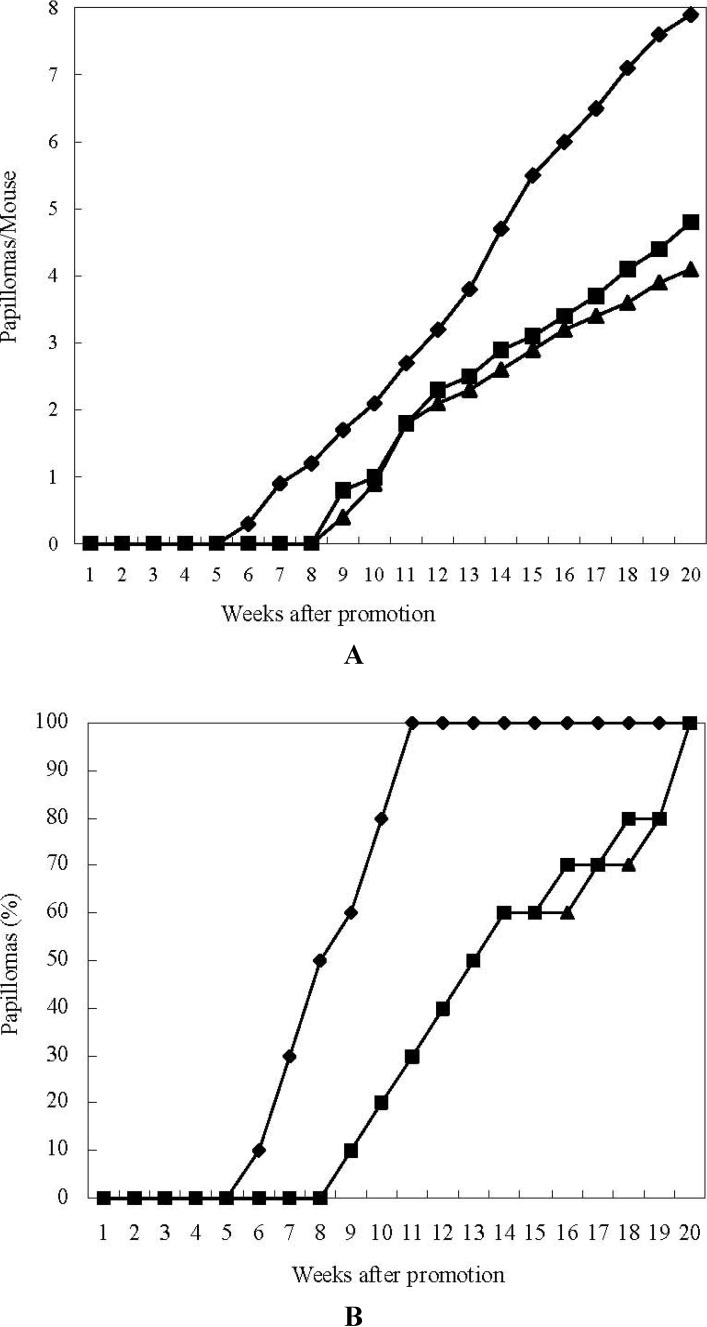
Inhibitory effects of astaxanthin and 15-nitroastaxanthin on 7,12-dimethylbenz [*a*]anthracene (DMBA) induced mouse skin carcinogenesis. (**A**) Average number of papillomas per mouse; (**B**) Percentage of mice bearing papillomas. ♦-♦ DMBA (390 nmol) + 12-*O*-tetradecanoylphorbol-13-acetate (TPA) (1.7 nmol); ■-■ Astaxanthin (85 nmol) + DMBA (390 nmol) + TPA (1.7 nmol); ▲-▲15-Nitroastaxanthin (85 nmol) + DMBA (390 nmol) + TPA (1.7 nmol).

Next, the inhibitory effects of astaxanthin and 15-nitroastaxanthin on two-stage mouse skin carcinogenesis [19] were investigated. The average number of papillomas per mouse and the incidence (%) of the mice bearing papillomas in the astaxanthin, 15-nitroastaxanthin, and control groups are shown in Figure 2.

When astaxanthin and 15-nitroastaxanthin were applied before each 12-*O*-tetradecanoylphorbol-13-acetate (TPA) treatment, they markedly delayed the formation of papillomas and reduced the average numbers of papillomas per mouse compared with the control group. Among them, 15-nitroastaxanthin showed slightly higher inhibitory activity than astaxanthin, as shown in Figure 2.

**Figure 3:**
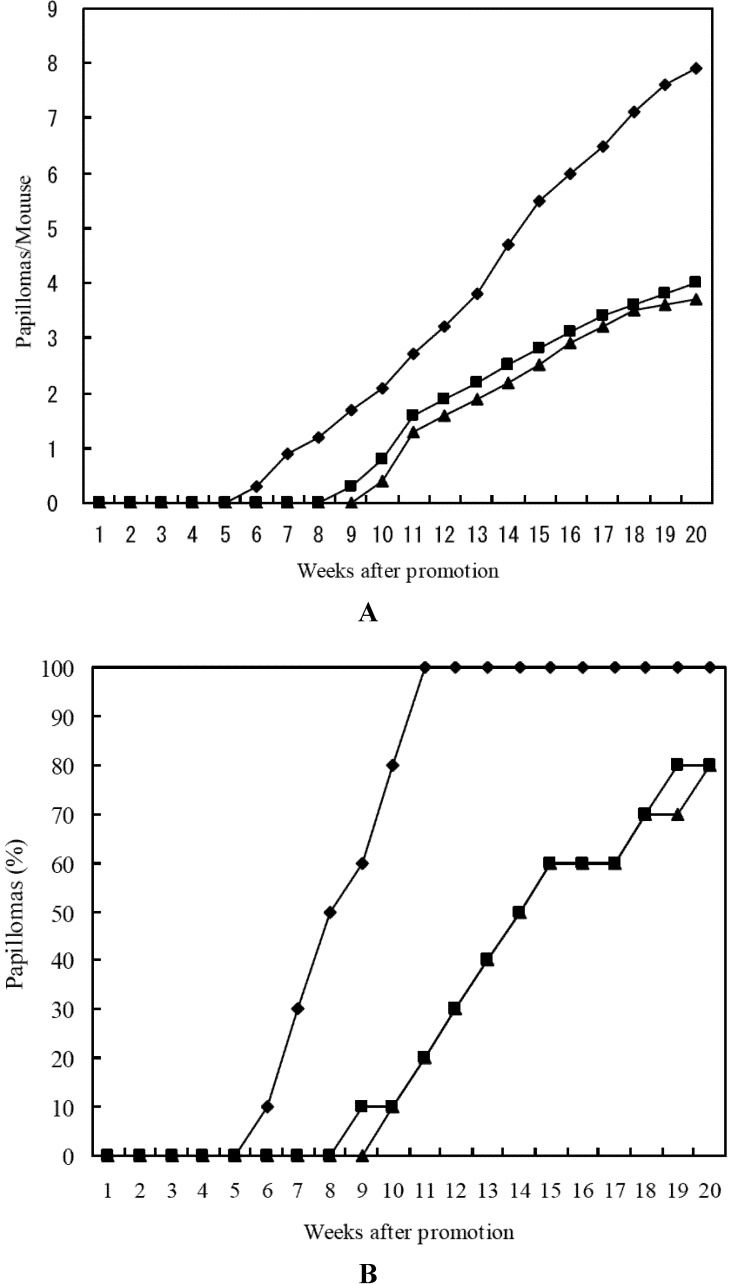
Inhibitory effects of lutein and 15-nitrolutein on DMBA induced mouse skin carcinogenesis. (**A**) Average number of papillomas per mouse; (**B**) Percentage of mice bearing papillomas. ♦-♦ DMBA (390 nmol) + TPA (1.7 nmol); ■-■ Lutein(85 nmol) + DMBA (390 nmol) + TPA (1.7 nmol); ▲-▲15-Nitrolutein (85 nmol) + DMBA (390 nmol) + TPA (1.7 nmol).

Similar results were obtained for lutein and 15-nitrolutein, as shown in Figure 3. 15-Nitrolutein showed slightly higher inhibitory activity than lutein. These results indicated that nitrocarotenoids themselves showed anti-carcinogenesis effect.

Previously, we reported that capsanthin and fucoxanthin could take up peroxynitrite through the formation of nitrocapsanthins and nitrofucoxanthins, respectively, and inhibit tyrosine nitration by peroxynitrite. Nitrocapsanthins and nitrofucoxanthins also exhibited anti-tumor-promoting activity. In the present study, similar results were obtained for astaxanthin and lutein [11]. Therefore, it was assumed that carotenoids could inhibit peroxynitrite-induced damages such as, oxidation of lipids and protein, DNA stanrard scission, and induction of carcinogenesis.

## 3. Experimental Section

### 3.1. Preparation of 15-Nitroastaxanthin and 15-Nitrolutein

15-Nitroastaxanthin and 15-nitrolutein were prepared by reaction of astaxanthin and lutein with peroxynitrite according to the method previously reported [4,6].

### 3.2. Inhibition of Tyrosine Nitration with Peroxynitrite by Carotenoids

Astaxanthin or lutein (0.2 mg) was dissolved in 1 mL of methanol (MeOH). L-Tyrosine (0.36 mg) was added to this solution. In the control group, L-tyrosine (0.36 mg) was dissolved in 1 mL of MeOH. Then, 20 μL of peroxynitrite (final concentration: 6.8 mM) was added to the solution. The same procedure was carried out for γ-tocopherol (0.15 mg), which was used as a positive control for this assay. The solution was subsequently allowed to react for 5 min at room temperature. After that, the nitrotyrosine content, a product of the reaction between tyrosine and peroxynitrite, was quantified using HPLC. The following HPLC systems were employed for the quantification of nitrotyrosine. Column: Develosil ODS-UG-5 (20.0 × 250 i.d. mm; mobile phase: phosphate-buffer (50 mM)/MeOH (97:3 v/v), flow rate: 1 mL/min, column temp: 40 °C, detection: 250 nm. Inhibition of the nitrotyrosine generated was measured by comparing with the absorbance of the sample solution (in MeOH).

### 3.3. Measurement of ^1^O_2_ Quenching Activity

^1^O_2_ quenching activity was examined by measuring methylene blue-sensitized photooxidation of linoleic acid [15]. Forty microliters of 0.05 mM methylene blue, 10 μL of 2.4 M linoleic acid with or without 40 μL carotenoid (final concentration 1–100 μM, each dissolved in EtOH) were added to micro glass vials (5.0 mL). The vials were tightly closed with a screw cap and a septum, and the mixtures were illuminated at 7000 lux at 22 °C for 3 h in corrugated cardboard. Then, 50 μL of the reaction mixture was removed and diluted to 1.5 mL with EtOH, and absorbance at 235 nm was measured to estimate the formation of conjugates dienes. The value in the absence of carotenoid was determined and ^1^O_2_ repression activity was calculated relative to this reference value. Activity is indicated as the IC_50_ value representing the concentration at which 50% inhibition was observed [15].

### 3.4. *In Vitro* Epstein-Barr Virus Early Antigen (EBV-EA) Activation Induction Effect

EBV genome-carrying lymphoblastoid cells (Raji cells) derived from Burkitt’s lymphoma were cultivated in RPMI-1640 medium with 10% fetal bovine serum (FBS). The Raji cells were incubated for 48 h at 37 °C in a medium containing *n*-butyric acid (4 nmol), TPA (32 pmol), and various amounts of test compounds. Smears were made from the cell suspension, and we employed an indirect immunofluorescence technique. Details of the in vitro assay for EBV-EA induction have been reported previously [18].

### 3.5. *In Vivo* Two-Stage Carcinogensis Assay on Mouse Skin Initiated by DMBA and Promoted by TPA

The animals (specific pathogen-free female ICR-6-week old mice) were divided into three experimental groups, each with 10 mice. The backs of the mice were shaved with surgical clippers, and they were treated topically with 7,12-dimethylbenz[*a*]anthracene (DMBA) (100 μg, 390 nmol) in acetone (0.1 mL) as the initiator. One week after initiation, papilloma formation was promoted twice a week by the application of TPA (1 μg, 1.7 nmol) in acetone (0.1 mL) to the skin. The control group received TPA treatment alone. Experimental groups received topical application of carotenoids (85 nmol) in acetone (0.1 mL), 1 h before the TPA treatment. The incidence and numbers of papillomas were monitored weekly for 20 weeks [11,19].

Experiments involving mice were conducted in accordance with Kanazawa University, Institute for Experimental Animals and use Committee Guidelines, under the jurisdiction of the Ministry of Education, Culture, Sports, Science and Technology.

## 4. Conclusions

Astaxanthin and lutein could take up peroxynitrite through the formation of nitroastaxanthin and nitrolutein, respectively, and inhibit tyrosine nitration by peroxynitrite. Furthermore, 15-nitroastaxanthin and 15-nitrolutein exhibited ^1^O_2_ quenching, anti-tumor-promoting, anti-carcinogenesis activities. Therefore, astxanthin and lutein may have the potential to reduce the risk of disease induced by reactive nitrogen species and prevent active nitrogen-induced disease.
